# Modern Contraceptive Utilization and Determinant Factors among Street Reproductive-Aged Women in Amhara Regional State Zonal Towns, North West Ethiopia, 2019: Community-Based Study

**DOI:** 10.1155/2020/7345820

**Published:** 2020-11-28

**Authors:** Wondimnew Gashaw Kettema, Getie Lake Aynalem, Ayenew Engida Yismaw, Ayenew Worku Degu

**Affiliations:** ^1^Department of Midwifery, Wollo University, Dessie City, Ethiopia; ^2^School of Midwifery, University of Gondar, Gondar City, Ethiopia

## Abstract

**Objective:**

Reproductive-aged women living on the street, with no doubt, are with lesser benefits of exercising their reproductive rights. Pregnancies from this marginalized population are likely to be unplanned, unwanted, and unsupported. The aim of this study, therefore, was to assess modern contraception utilization and associated factors among street reproductive-aged women in Amhara regional state zonal towns.

**Method:**

A community-based cross-sectional study was conducted among street reproductive-aged women in Amhara regional state zonal towns. A single population proportion formula was used to calculate the sample size, a similar literature-based tool adaptation was done, and a semistructured, pretested sectioned questionnaire was used. Cluster sampling technique was used to reach the study participants. Data was entered into Epi Info version 7 and exported to SPSS version 23 for analysis. A multivariable logistic regression model was fitted to control the possible effect of confounders, and finally, the independent variables were identified on the basis of OR, with 95% CI and *p* values less than 0.05.

**Results:**

604 street reproductive-aged women were interviewed in the study which make the response rate 94.2%. The study revealed that current modern contraceptive utilization among the study participants was found to be 38.9%. Having history of pregnancy in street life (AOR = 1.70, 1.1-2.7), having three or more live children (AOR = 6.4, 2.0-20.4), undesiring to have additional children in the future (AOR = 2.7, 1.4-5.1), mentioning three to four (AOR = 2.2, 1.5-3.3) and five or more modern contraceptive types (AOR = 5.5, 1.4-21.0), and discussion with sexual partners for contraceptive use (AOR = 6.6, 4.3-10.1) were variables significantly associated with modern contraceptive utilization. Modern contraceptive utilization among the street reproductive-aged women was low. Authors suggest that awareness creation and male partner involvement in the maternal services may be important to increase contraceptive utilization.

## 1. Introduction

Contraceptives are one of the most important health interventions of the 21^st^ century which is crucial in reducing rapid population growth and improving women and family health [1]. For that, the Sustainable Development Goals targeted to expand access to family planning under its target point of 3.7 [2].

Family planning saves lives and improves quality of life of women, children, and family at large. It also prevents pregnancy-related health risks in women, infant mortality, sexually transmitted infection (STI) and HIV/AIDS, and adolescent pregnancies and slows population growth [3, 4]. Reports showed that one-quarter of maternal deaths could be averted by appropriately using contraception [5].

According to a 2015 worldwide report, unmet need for family planning was 12% among married women. Sub-Saharan Africa's unmet need for family planning (22%) is nearly twice that of other continent's report (7-15%). Similarly, in Ethiopia, 22% of married women had an unmet need for family planning [6, 7].

Street women are a high-risk group for unintended pregnancies [8]. Almost three-fourths (73%) of homeless women reported that their pregnancies were unintended according to the study done in Los Angeles, and half of them had a history of STD and pelvic inflammatory diseases in that study [9].

Moreover, homelessness during pregnancy increases the risk of low birth weight, small size for gestational age, and preterm birth. Although these women have strong desires to prevent pregnancy, reports show that pregnancy rates among homeless are much higher than rates among women who are housed [10–13].

Another study done in Montreal, Canada, among street women showed that there were high unintended pregnancies due to the failure of contraceptive method, carelessness for future life, drug use, and sexual abuse in street life [14].

Despite Ethiopia's contraceptive prevalence rate increasing from 29% in 2011 to 35% in 2016, the target was 65% by 2015 which was unachieved. Previous different researches showed that women with low monthly income and unable to read and write had reported low modern contraceptive utilization as compared to rich and educated women [3, 6, 15].

In the first half of 2018, Ethiopia has the highest number of internally displaced people than any other country, and homelessness and street residence is an escalating national problem [16].

The Ethiopian health sector gives emphasis to achieve the goal of the Health Sector Transformation Plan (HSTP) which is universal health coverage by strengthening primary health care, but street women living in informal settlements are likely excluded from formal public services, including contraceptive services [17].

A study done in Addis Ababa, capital city of Ethiopia, showed that about 36.9% of street women experienced unintentional pregnancy [18], and another study conducted in Bahir Dar town, capital city of the Amhara regional state, revealed a high rate of female rape (24.3%) of whom 19.1% ended with unwanted pregnancy [19].

Therefore, this study was conducted to assess modern contraceptive utilization and associated factors among street reproductive-aged women in Amhara regional state zonal towns.

## 2. Methods

### 2.1. Study Design and Setting

A community-based cross-sectional study was conducted among street reproductive-aged women in Amhara regional state zonal towns from August 15 to October 30, 2019. The Amhara regional state is one of the 10 Ethiopian national regional states. Its capital city is Bahir Dar. This region has 11 administrative zones and 140 Woredas, 80 hospitals (5 referral, 2 general, and 73 primary), 847 health centers, and 3,342 health posts according to the available data [20, 21]. In the 2018 annual report of the Amhara National Regional State Bureau of Labor and Social Affairs, there were 2024 street reproductive-aged women in Amhara regional state [22].

### 2.2. Sample Size Determination and Techniques

We used a single population proportion formula using the following assumptions: proportion (*p*) 34.3% (taken from street women modern contraceptive utilization done in Bahir Dar and Gondar cities) [23], 95% confidence level, 5% margin of error, 1.5 design effect, and 10% nonresponse rate. That is, *n* = (Z*α*/2)^2^*p*(1 − *p*)/*d*^2^, *n* = 570.

Cluster sampling technique was used to select study participants. We used the previously divided eleven zonal towns of which five were selected by lottery method that were Dessie, Debre Tabor, Bahir Dar, Debre Markos, and Woldia towns. Then, all street women in the selected zonal towns who fulfilled the inclusion criteria and were available during the data collection period were included in the study. For this sampling procedure, we used the Amhara regional state Labor and Social Affairs annual report of 2010. Since street women are mobile and have no definite addresses, the probability of getting interviewed street women on other sites is high. To avoid interviewing more than once, participants were asked whether or not they were previously interviewed for the purpose of modern contraceptive utilization within the period of data collection. The populations for each zonal town were as follows: Bahir Dar (186), Dessie (182), Debre Markos (106), Debre Tabor (86), and Woldia (81) in which all totaled a sample size of 641 from whom 604 participated in the study.

### 2.3. Operational Definitions

- *Modern contraceptive methods*: the product or medical procedure that interferes with reproduction from acts of sexual intercourse which includes female sterilization, male sterilization, pills, the intrauterine device (IUD), injectable, implants, and condom [24]
- *Current modern contraceptive use*: when a woman uses any of the modern contraception methods during data collection [6]
- *Street women*: women who make their living on the street by begging, sleeping on the street or road sides which include both off-street women and on-street women [25]
- *On-street women*: women who had no formal home (homeless) who live and sleep on streets, verandas, and balconies [25]
- *Off-street women*: women who have houses to go for sleep at night while making their living on the street or begging for money on the street and returning to their formal home at night for sleeping [25]

### 2.4. Data Collection Tool and Procedure

Data were collected using a semistructured questionnaire which was adapted from similar studies. The questionnaire was prepared in English and translated to local language (Amharic) for data collection and then translated back to English to keep the consistency of the questionnaire. Data were collected around churches in the morning, mosques on Friday lunch time, main street, and bus stations (sites where street women were sitting begging in queues).

### 2.5. Data Quality Control

A pretest was conducted in Gondar town on a 5% sample size prior to the actual data collection. Training was given for fifteen data collectors and five supervisors which means three data collectors and one supervisor for each town. At the end of each day, the questionnaires were reviewed and checked for completeness, accuracy, and consistency by the supervisor and principal investigator, and corrective discussion was undertaken with data collectors.

### 2.6. Data Processing and Analysis

The data were entered into Epi Info version 7 and exported to SPSS version 23 for analysis. A multivariable logistic regression model was fitted to control the possible effect of confounders, and finally, the independent variables which had associations with the outcome variable were identified on the basis of OR, with 95% CI and *p* values less than 0.05.

## 3. Results

### 3.1. Sociodemographic Results

A total of 604 street reproductive-aged women were interviewed which gives a response rate of 94.2%. Half (50.7%) of the respondents were in the age group of 35-49 years with the median age of 35 years (IQR: 10 years). From total respondents, 382 (63.2%) had reported that they had sexual partners. Around one in eight participants, 12.6%, were on-street (homeless) women (Table 1).

### 3.2. Reproductive and Other Health-Related Characteristics

More than half, 340 (56.3%), of the respondents reported that they had at least one experience of pregnancy in street life of whom 203(59.7%) had at least one unplanned pregnancy. One in ten respondents, 70 (11.6%), reported that they had a history of rape in their street life (Table 2).

### 3.3. Magnitude of Modern Contraceptive Utilization among the Street Women

Five in eight (61.8%) of the study participants reported that they had used modern contraceptives in their past lifetime. Out of 382 study participants who had a sexual partner, half (49.5%) reported that they had discussions with their partner to use modern contraceptives. Regarding current utilization of modern contraceptives, about 235 (38.9%) (95% CI, 35.3-42.7%) participants were using currently. Among those who were not using modern contraceptives currently, half (50.4%) had revealed that they did not have a plan to use in the future too.

### 3.4. Factors Associated with Modern Contraceptive Utilization among the Study Participants

Crudely associated variables were age, having sexual partner, family size, having history of pregnancy in street life, number of current children, time plan for having child in the future, number of contraceptives mentioned by the participant, and having discussions with sexual partners.

From the crudely associated variables, having history of pregnancy in street life, reporting three or more current children, undesiring for more children in the future, mentioning a larger number of contraceptives, and discussions with sexual partners for contraceptive use were found to be significantly associated with modern contraceptive utilization in the multivariable logistic regression analysis (Table 3).

## 4. Discussion

The main objectives of this study were to assess modern contraceptive utilization and associated factors among street reproductive-aged women in Amhara regional state zonal towns. According to the finding of this study, 38.9% of the participants were currently using modern contraceptives.

This current modern contraceptive utilization is comparable with findings among slum dwellers in Nairobi, Kenya (37.9%), and street beggar women in South Ethiopia (37.4%) [26, 27].

But this finding is lower than findings in Dehradun city, India (56.8%), an urban slum in Pune (68.5%), and urban slum dwellers of Bangladesh (53.2%) [26–30]. This discrepancy could be due to previous studies including traditional methods of contraceptives like the lactational amenorrhea method (LAM) which might increase the prevalence and, in addition, sociodemographic differences among the study participants.

On the other hand, this finding is relatively higher than other findings done on Dhaka street dwellers (34.5%); Rajasthan slum dwellers (24%); Eritrean refugee camps (26%); migratory/slum dwellers of Karnataka, India (32%); and North West Ethiopia (34.3%) [23, 31–34]. It could be due to pregnant women being included previously which could decrease the prevalence of contraceptives and the time gap between the studies.

The study has revealed that history of pregnancy in street life was one of the positive predictor variables. Street women who had a history of pregnancy in street life were 1.7 times more likely to use modern contraceptives when compared with their counterparts (AOR = 1.7, 95% CI: 1.10, 2.7). This finding is supported by the finding in street women in Southern Ethiopia [27]. Taking a lesson from previous sufferings of labor and child care including feeding during difficult street life might be the possible reasons to utilize modern contraceptives.

The number of current children was another predictor variable. Street women who have three or more live children currently were 6.4 times more likely to use modern contraceptives as compared to those who have no live child (AOR = 6.4, 95% CI: 2.0, 20.4). This result is supported by the findings in slums in Bangladesh, Kenya, and India [26, 29, 30]. It could be that such women might have achieved their desired number of children and might be likely to use contraception.

Street women who did not want to have children in the future were 2.7 times more likely to use modern contraceptives as compared to those who wanted children within two years (AOR = 2.7, 1.4-5.1). This is in line with literatures from Debre Birhan district and North Shewa zone [35, 36]. It could be due to the fact that if women do not want to get pregnant, it is likely that they might use contraception.

Mentioning modern contraceptive methods was also one of the positive predictor variables for modern contraceptive utilization. Women who listed down five and above modern contraceptive types were more likely to use the modern contraception when compared with those who listed down two or less modern contraceptive types (AOR = 5.5, 1.4-21.0). The result is supported by findings from Debre Birhan district and Tigray region [35, 37]. This could be due to women getting their preferences among the options they mentioned and being likely to use them. This indicates that awareness creation for this population is important for modern contraception utilization.

The odds of using modern contraceptives for women who had discussions with their sexual partners were 6.6 times more likely to use modern contraceptive methods than the counterparts (AOR = 6.6, 4.3-10.1). This is in line with studies conducted in Bale zone, Melokoza, Tigray region, rural Dembia, and North Shewa [36–40]. It could be due to the fact that discussion might increase awareness about the value of contraception and initiated the study participants to use contraceptives.

## 5. Conclusion

The study showed that modern contraceptive utilization among street women in Amhara regional state zonal towns was low when compared with the targeted contraceptive prevalence rates of Ethiopia and Amhara regional state.

Having a history of pregnancy in street life, high number of current children, undesiring to have children in the future, mentioning more number of contraceptives, and having discussions with sexual partner for contraceptive use were predictor variables of modern contraceptive utilization among the study participants.

Reasons mentioned by the study participants for not using modern contraceptives currently were knowledge on modern contraceptives, fear of side effects, male partner opposition, unmet need on preference of modern contraceptive, inconvenient service provision time, and inaccessibility of the services.

## Figures and Tables

**Table 1:** Sociodemographic characteristics of respondents in Amhara regional state zonal towns, North West Ethiopia, 2019 (*N* = 604).

Variable	Frequency (*n*)	Percentage (%)
*Address*		
Bahir Dar	177	29.3
Debre Markos	102	16.9
Debre Tabor	83	13.7
Dessie	164	27.2
Woldia	78	12.9
*Age (years)*		
15-24	52	8.6
25-34	246	40.7
35-49	306	50.7
*Ethnicity*		
Amhara	596	98.7
Other^1^	8	1.3
*Religion*		
Orthodox	537	88.9
Muslim	67	11.1
*Educational status*		
Cannot read and write	468	77.5
Can read and write only	54	8.9
Elementary school or above	82	13.6
*Have sexual partner*		
Yes	382	63.2
No	222	36.8
*Residence before street life*		
Urban	352	58.3
Rural	252	41.7
*Current residence*		
Urban	555	91.9
Rural	49	8.1
*Sleeping place at night*		
On street	528	87.4
Off street	76	12.6
*Knows the place where the health service is*		
Yes	573	94.9
No	31	5.1
*Time it will take to arrive at nearby health facility on foot (N* = 573)		
≤30 minutes	525	91.6
>30 minutes	48	8.4
*Family size*		
1	51	8.4
2-3	312	51.7
≥4	241	39.9
*Duration of street life (years)*		
0.5-5	367	60.8
6-10	175	29.0
11-15	44	7.2
>15	18	3.0

^1^Includes Tigray and Addis Ababa.

**Table 2:** Reproductive and other health-related characteristics of respondents in Amhara regional state zonal towns, North West Ethiopia, 2019 (*N* = 604).

Variable	Frequency (*n*)	Percentage (%)
*Pregnant in street life*		
Yes	340	56.3
No	264	43.7
*Number of pregnancies in street life (N* = 340)		
1	202	59.4
2	90	26.5
≥3	48	14.1
*Plan for previous street pregnancies (N* = 340)		
Planned	137	40.3
Unplanned	203	59.7
*Number of alive children*		
0	48	8.0
1-2	339	56.1
≥3	217	35.9
*Time elapsed since last child birth/termination (months) (N* = 553)		
0-17	139	25.1
18-59	289	52.3
≥60	125	22.6
*Desire to have child in the future*		
Yes	234	38.7
No	370	61.3
*Time of plan to have child in the future (N* = 234)		
Within two years	95	40.6
After two years	139	59.4
*Raped in street life*		
Yes	70	11.6
No	534	88.4
*Number of rapes in street life (N* = 70)		
1	44	62.9
2-4	26	37.1
*Have chronic illnesses/disabilities*		
Yes	304	50.3
No	300	49.7
*Types of chronic illnesses/disabilities (N* = 304*)^2^*		
Vision impairment	85	28.0
Problem on leg/hand	100	32.9
Nonspecified internal problem	60	19.7
HIV/AIDS	47	15.5
Other^3^	19	6.3

^2^More than one response is possible. ^3^Includes diabetes mellitus, epilepsy, hemorrhoid, hypertension, liver cancer, pelvic organ prolapse, and tuberculosis.

**Table 3:** Bivariable and multivariable analysis of factors associated with modern contraceptive utilization among street reproductive-aged women in Amhara regional state zonal towns, North West Ethiopia, 2019 (*n* = 604).

Variable	Modern contraceptive use		COR (95% CI)	AOR (95% CI)
	Yes	No		
*Age*				
15-24	15	37	1	1
25-34	107	139	1.9 (1.0-3.7)^∗^	2.1 (0.9-4.7)
35-49	113	193	1.4 (0.8-2.7)	1.0 (0.4-2.2)
*Have sexual partner*				
Yes	177	205	2.4 (1.7-3.5)^∗∗^	1.17 (0.7-1.9)
No	58	164	1	1
*Family size*				
Alone	10	41	1	1
2-3	124	188	2.7 (1.3-5.7)^∗∗^	1.2 (0.4-3.0)
≥4	101	140	2.9 (1.4-6.1)^∗∗^	0.5 (0.2-1.3)
*History of pregnancy in street life*				
Yes	156	184	2.0 (1.4-2.8)^∗∗^	1.7 (1.1-2.7)^∗^
No	79	185	1	1
*Number of alive children*				
No living children	5	43	1	1
One to two	127	212	4.1 (1.7-10.0)^∗∗^	2.1 (0.7-6.3)
Three and above	103	114	6.3 (2.6-15.5)^∗∗^	6.4 (2.00-20.4)^∗∗^
*Desire time of child*				
Within two years	24	71	1	1
After two years	55	84	1.9 (1.1-3.4)^∗^	1.9 (0.9-3.8)
No more child wanted	156	214	2.2 (1.3-3.6)^∗∗^	2.7 (1.4-5.1)^∗∗^
*Number of contraceptives mentioned*				
≤2	107	253	1	1
3-4	121	111	2.6 (1.8-3.6)^∗∗^	0.2 (0.5-3.3)
≥5	7	5	3.3 (1.0-10.7)^∗^	5.5 (1.4-21.0)^∗^
*Discussion with sexual partner*				
Yes	128	61	6.2 (4.0-9.6)^∗∗^	6.6 (4.3-10.1)^∗∗^
No	49	144	1	1

Keys: ^∗^*p* value < 0.05 and ^∗∗^*p* values < 0.01.

## Data Availability

The datasets used and/or analyzed during the current study are available from the corresponding author on reasonable request.

## Additional Points

*Limitations of This Research*. We might not have possibly reached in the study participants who might be severely disabled and spending their time at their formal home, and our study lacks comparison with the general population.
